# Measurement prediction and power analysis for fNIRS and DOT

**DOI:** 10.1162/IMAG.a.1289

**Published:** 2026-07-01

**Authors:** Eli Bulger, Jiaming Cao, Abigail L. Noyce, Barbara G. Shinn-Cunningham, Jana M. Kainerstorfer

**Affiliations:** Department of Biomedical Engineering, Carnegie Mellon University, Pittsburgh, PA, United States; Neuroscience Institute, Carnegie Mellon University, Pittsburgh, PA, United States; Department of Psychology, Carnegie Mellon University, Pittsburgh, PA, United States; Department of Electrical and Computer Engineering, Carnegie Mellon University, Pittsburgh, PA, United States

**Keywords:** functional near-infrared spectroscopy, diffuse optical tomography, power analysis, forward modeling, cluster-based permutation testing, experimental design

## Abstract

Functional near-infrared spectroscopy (fNIRS) and diffuse optical tomography (DOT) are valuable neuroscience tools, but their feasibility for some research questions can be uncertain due to interacting anatomical, measurement, and design limitations. Here, we present an experimentally informed computational framework for *a priori* power analysis that combines within- and between-subject variability estimates with forward-model–based estimates of task-evoked absorption changes to predict measurements. Cluster-based permutation tests applied to these measurements estimate statistical power for signal and contrast detection across sample sizes and stimulus block counts. We demonstrate how channel density and cortical source features—depth, size, spatial separation, and location—influence statistical power. Our approach reframes the physical constraints of fNIRS and DOT into interpretable design guidance and can be adopted to support principled power planning across diverse experimental designs.

## 1 Introduction

Near-infrared spectroscopy (NIRS) is a non-invasive imaging technique that uses the scattering and absorption of near-infrared light to probe biological tissue. Functional NIRS (fNIRS) estimates hemodynamic responses by leveraging the differential absorption of near-infrared light by oxygenated and deoxygenated hemoglobin (HbO and HbR) (Hillman, 2007). Traditional implementations rely on sparse optode arrays and offer limited spatial resolution, but higher-density configurations enable diffuse optical tomography (DOT), a volumetric extension of fNIRS that enhances cortical source localization and spatial fidelity (Zeff et al., 2007). In high-density DOT, increased channel density can substantially improve spatial resolution, in some cases approaching that reported for functional magnetic resonance imaging (fMRI) (Cui et al., 2011; Eggebrecht et al., 2012; White & Culver, 2010).

While DOT offers substantial improvements in spatial resolution and reconstruction accuracy over traditional fNIRS, its practical deployment faces challenges. These include predominant sensitivity to the superficial cortex (≈ 1–1.5 cm depth), susceptibility to spontaneous hemodynamics, physiological and motion confounds, probe–head geometry/coverage and optode–scalp coupling (e.g., hair/skin effects, short-separation availability), and population-dependent optical/physiological variability (Yücel et al., 2021). The resulting uncertainty in signal and noise characteristics can make feasibility ambiguous for a given paradigm. This uncertainty is especially challenging for teams new to fNIRS/DOT and increases the barrier to implementation. In such cases, *a priori* power analyses help determine whether a DOT study is likely to yield detectable effects. These analyses require predicting the spatial distribution and magnitude of the expected response and the within- and between-subject variance at the channel level.

Extensive prior work has attempted to predict the signals and sensitivity of fNIRS and DOT using tools that model photon propagation, such as Monte Carlo simulations and finite element modeling (e.g., NIRFAST (Dehghani et al., 2008)). These models enable forward prediction of channel-level data and inverse prediction of cortical sources. This body of work provides a strong foundation for understanding the physical limitations of fNIRS and DOT. Complementary efforts have focused on optimizing optode placement to enhance sensitivity to specific cortical regions (Brigadoi et al., 2018; Cao et al., 2022; Zimeo Morais et al., 2018) and on improving depth sensitivity using both model-informed (Chiarelli et al., 2016; Tremblay et al., 2018) and data-informed (Qin et al., 2024) strategies. Similar to fMRI literature (Chen et al., 2017), many studies in fNIRS/DOT literature report results in arbitrary or relative units (e.g., percentage sensitivity), rather than in physiologically meaningful units (e.g., *μ*M concentration changes). Although forward and inverse models can convert absorption changes (Δ*μ_a_*) into *μ*M, these predictions are rarely translated into concrete design guidelines—such as recommended sample sizes or block counts. This is of particular importance in experimental contexts where the underlying absorption changes may be near the threshold of detectability and are not already well established. As a result, the feasibility of measuring real physiological changes is often not fully quantified in practical applications of fNIRS and DOT.

Another line of work has characterized signal variance and noise in fNIRS and DOT, which is necessary for statistical power analyses. In the spatial dimension, tissue structures vary within and between subjects, yielding different DOT contrast-to-noise ratios in different channel locations (Perdue et al., 2012). Across time, fNIRS measurements can exhibit within-subject noise levels similar to those reported for fMRI, but often show higher across-subject variability (Minati et al., 2011), even after improving consistency via global physiological regression and measurement of optode placement (Novi et al., 2020; Sinfield et al., 2025). Related work has focused on applying noise reduction techniques—such as short-separation channel regression and other physiological signal modeling—to improve data quality (Saager & Berger, 2005; Scholkmann et al., 2022).

Due to the significant variability in signal quality across cortical locations or experimental manipulations, the practical feasibility of these imaging tools remains difficult to evaluate. Statistical methods to support such decisions are scarce, primarily focusing on analysis-time inference as opposed to design-time planning (Hassanpour et al., 2014; Tak & Ye, 2014). Here, we present an approach to conduct *a priori* power analyses of an fNIRS or DOT design. We first perform forward modeling using changes in absorption coefficients estimated from prior DOT measurements. Using within- and between-subject variability estimated from empirical measurements, measurement predictions are integrated into a cluster-based permutation test power analysis that accounts for spatial correlations and controls family-wise error rates (Groppe et al., 2011; Maris & Oostenveld, 2007). Our pipeline is thus able to estimate the feasibility of using fNIRS and DOT to target specific cortical regions in defined experimental contexts. We apply our methodology in simulation to illustrate how statistical power is affected by cortical source depth, size and spatial separation of contrasted sources, source location, and channel density.

## 2 Methods

**Fig. 1.**
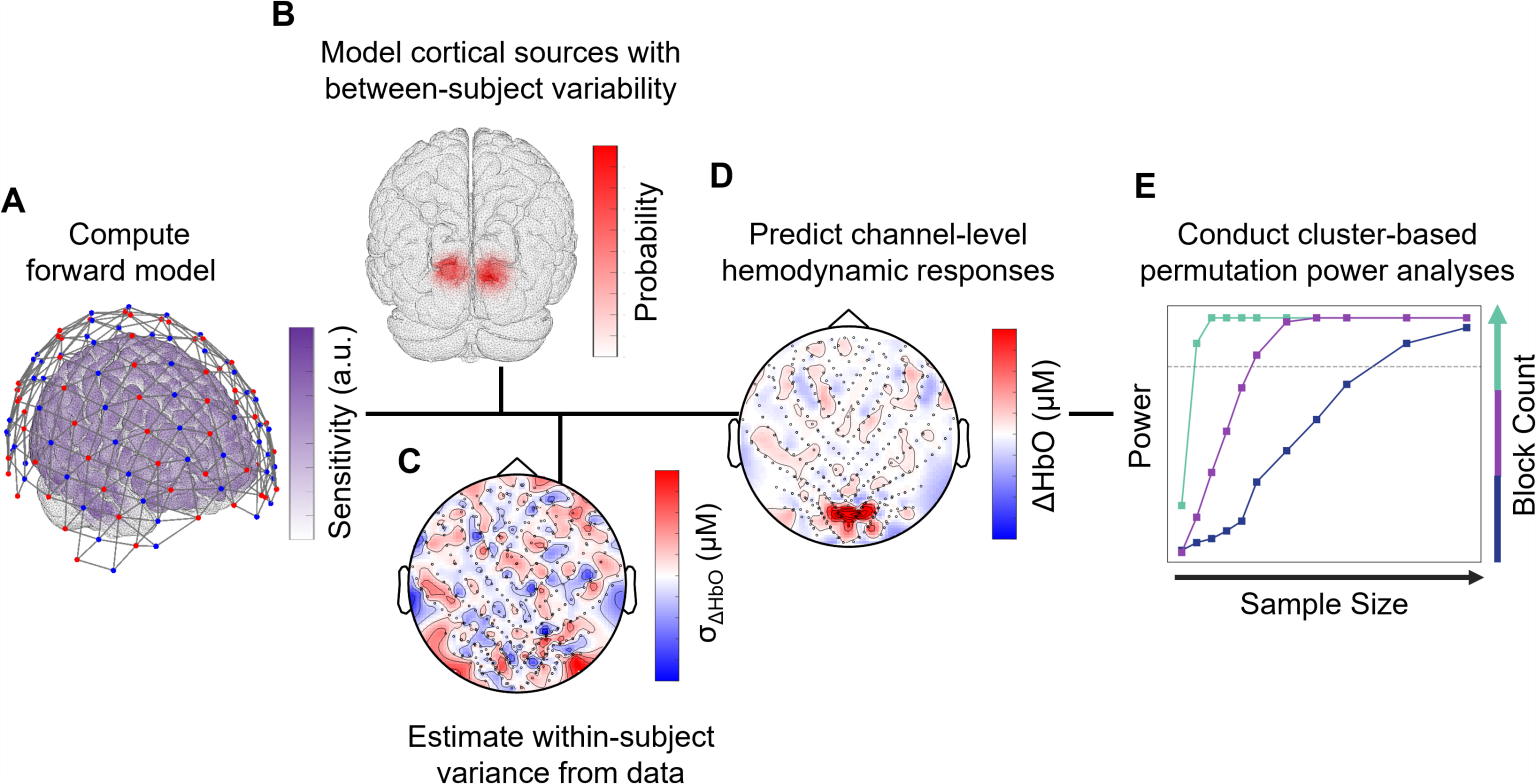
Schematic of steps to conduct *a priori* power analyses for experiments using fNIRS or DOT. (A) A forward sensitivity matrix is computed using NIRFAST, relating absorption changes in a five-layer head mesh (derived from the ICBM 2009c asymmetric template) to an optode montage defined in NIRSite (NIRx). Absorption magnitudes to be used in following simulations can be estimated from experimental data using this forward model. The panel illustrates the optode montage and cortical mesh overlaid with normalized flatfield sensitivity (purple), sources (red), detectors (blue), and channels (grey lines). (B) Cortical sources are modeled with physiologically plausible between-subject variability, represented here as a probabilistic map of exemplar source locations across simulated subjects. (C) Within-subject variance is estimated from channel-level ΔHbO time series as a function of block count, illustrated as channel-wise variability. (D) Task-evoked cortical activations are forward-modeled and combined with simulated within-subject variability to generate predicted channel-level ΔHbO measurements. (E) Simulated datasets are analyzed using cluster-based permutation testing to estimate statistical power as a function of sample size and block count. Block count is represented, in ascending order, by blue, purple, and cyan lines.

We report a framework for computing statistical power in fNIRS and DOT measurements (Fig. 1), integrating forward modeling, absorption magnitude estimation, variability modeling, measurement prediction, and power analysis across experimental design parameters.

The first step in our approach is to estimate absorption coefficient magnitudes within the cortex. While DOT can effectively localize cortical sources, the computed absorption change magnitudes within these sources, which are needed to estimate hemoglobin concentration changes, are often uncertain. To overcome this, we used empirical measurements from visual checkerboard wedge stimulation (Cao et al., 2023) and then performed DOT reconstruction to determine the location of activation. Using forward modeling, we then varied the absorption coefficient within that reconstructed space to minimize the difference between the predicted and measured channel-level data.

### 2.1 Experimental dataset

Data were recorded from 16 subjects during checkerboard wedge stimulation in alternating visual hemifields (25 blocks: 10 s fixation, 15 s stimulation, 25 s rest). All experimental procedures were approved by the Institutional Review Board of Carnegie Mellon University; written informed consent was obtained from all subjects. The DOT system (NIRx NIRSport2) provided 76 channels (24 sources, 21 detectors) over the occipital cortex at 5.1 Hz; source–detector distances ranged from 17.3–38.2 mm (mean 26.4 mm, SD 5.1 mm). Measurements were acquired at 760 and 850 nm. Raw intensity was converted to optical density (ΔOD = $-log10\left(I/I_{0}\right)$ with *I*_0_ being the average intensity per channel) and high-pass filtered at 0.01 Hz (4th-order Butterworth). Motion was mitigated with temporal derivative distribution repair (TDDR), a robust regression on the temporal derivative that identifies large, rapid deviations and downweights them during reconstruction (Fishburn et al., 2019), and a custom spike removal step that detects short motion spikes via a sliding-window sum of squared temporal differences and replaces flagged segments with linear interpolation between pre/post endpoints. We then applied a general linear model (GLM) to the ΔOD time series. Task-related regressors included one for visual stimulation blocks, modeled by stimulus boxcars convolved with a modeled hemodynamic response function; nuisance regressors included accelerometer-based motion parameters (when available; for 2 subjects, motion was approximated as the average of optical density data across channels), and low-order polynomials (constant and linear terms) to model baseline drift. Channel-wise, task-related regressor weights (β) at 760 nm and 850 nm were carried forward to enable absorption magnitude estimation.

### 2.2 Absorption magnitude estimation

To translate channel-wise task estimates into physically meaningful absorption changes, we back-projected them into grey matter via spatially-variant Tikhonov regularization (Dehghani et al., 2009; Eggebrecht et al., 2012), yielding a provisional Δ*μ_a_* map for each wavelength. We estimated the focal extent of each task-evoked source (one per hemisphere) as a full-width half-maximum (FWHM) using the 850 nm Δ*μ_a_* map, which exhibited the highest task-evoked contrast, to provide an empirical reference for subsequent simulations. For each hemisphere, FWHM was defined from the half-maximum region (≥50% of the hemispheric peak) by identifying its principal axes and measuring the width of the projected node coordinates along those axes using robust percentile bounds. Full methodological details are provided in Supplementary Information Section I. A cortical ΔHbO map derived from the wavelength-specific Δ*μ_a_* maps and extinction coefficients is visualized in Supplementary Figure S1 for reference.

The Δ*μ_a_* maps served as spatial priors for forward modeling and matching modeled and experimental data. We generated a five-layer head model (scalp, skull, cerebrospinal fluid (CSF), grey matter, white matter) by segmenting the ICBM 2009c Non-linear Asymmetric template in 3D Slicer. The binary masks were converted with iso2mesh into a tetrahedral finite-element mesh containing 372 703 nodes—145 806 located in grey matter—and 2 223 469 tetrahedra. Layer- and wavelength-specific optical properties (absorption *μ_a_* and reduced scattering $\mu_{s}^{'}$) at 760 nm and 850 nm were assigned from published sources and are shown in Table 1 (Bevilacqua et al., 1999; Custo et al., 2006; Strangman et al., 2002). The refractive index of each tissue layer was set to 1.4.

**Table 1.** Absorption (*μ_a_*) and reduced scattering ($\mu_{s}^{'}$) coefficients assigned to head mesh.

	760 nm		850 nm	
Tissue	*μ_a_* (mm ^− 1^)	$\mu_{s}^{'}$ (mm ^− 1^)	*μ_a_* (mm ^− 1^)	$\mu_{s}^{'}$ (mm ^− 1^)
White matter	0.0167	1.1908	0.0208	1.0107
Grey matter	0.0180	0.8359	0.0192	0.6726
CSF	0.0040	0.3000	0.0040	0.3000
Skull	0.0116	0.9400	0.0139	0.8400
Scalp	0.0170	0.7400	0.0190	0.6400

After defining the head model and optode montage (i.e., source-detector placement), we employed NIRFAST (Dehghani et al., 2008), which we use to solve the diffusion approximation to the radiative transport equation on our finite-element head mesh. The solution is in the form of the forward matrix, $J_{lnI}^{\left(\lambda \right)}$, which relates small absorption perturbations at each mesh node to changes in the log-intensity measured at each source–detector channel. We use $J_{lnI}^{\left(\lambda \right)}$ for two purposes: 1) to compute flatfield sensitivity maps visualizing the spatial sensitivity of our montage and 2) to construct a spectral Jacobian that enables prediction of absolute magnitude of ΔHbO and ΔHbR on the channel level (Fig. 1A).

We compute flatfield sensitivity by applying $J_{lnI}^{\left(\lambda \right)}$ to a uniform absorption distribution over grey matter and reconstructing the resulting channel predictions with spatially-variant Tikhonov regularization. To compute the spectral Jacobian, we stack $J_{lnI}^{\left(\lambda \right)}$ for 760 and 850 nm wavelengths (the wavelengths used in our experiments) into a block-diagonal Jacobian,

$$\begin{array}{c} J_{\text{ln}I}=\left(\begin{array}{cc} J_{\text{ln}I}^{\left(760\right)} & 0_{C\times N}\\ 0_{C\times N} & J_{\text{ln}I}^{\left(850\right)} \end{array}\right)\in {\mathbb{R}}^{2C\times 2N} \end{array}$$ (1)

where C is the number of channels and N the number of mesh nodes. We then define the 2 × 2 molar-extinction matrix, *E*, from tabulated values (Prahl, 1998), and apply its inverse independently to each channel so that the stacked Jacobian maps absorption changes in grey matter to channel-level ΔHbO and ΔHbR rather than wavelength-specific channel measurements.

$$\begin{array}{l} J_{\text{spec}}=\left(E^{-1}⊗I_{C}\right)J_{\text{ln}I}\in {\mathbb{R}}^{2C\times 2N} \end{array}$$ (2)

Each channel row is then normalized by its effective one-way pathlength, defined as half the two-way pathlength given by the row sum of *J_μ_*, so that ${\tilde{J}}_{\text{spec}}$ yields channel-level ΔHbO and ΔHbR in concentration units. This formulation yields hemoglobin changes comparable to those obtained using separate wavelength-specific forward modeling followed by the modified Beer–Lambert law (MBLL). In contrast to that two-step approach, in which pathlength is handled implicitly through a differential pathlength factor (DPF) term, our method incorporates pathlength normalization and spectral conversion within a single operator, thereby enforcing geometry- and channel-specific pathlengths and explicitly constraining HbO/HbR separation through the extinction coefficient matrix.

Applying ${\tilde{J}}_{\text{spec}}$ to absorption changes via a stacked Δ*μ_a_* vector yields the following,

$${\bar{J}}_{spec}\left(\begin{array}{c} \Delta \mu_{a}^{\left(760\right)}\\ \Delta \mu_{a}^{\left(850\right)} \end{array}\right)=\left(\begin{array}{c} \Delta HbO\\ \Delta HbO \end{array}\right)\in {\mathbb{R}}^{2C}$$ (3)

where the first *C* entries are channel-wise ΔHbO and the next *C* are ΔHbR . Finally, we iteratively scale Δ*μ_a_* in the forward model—using an L-curve optimized regularization that encourages a 3:1 HbO-to-HbR ratio—until the least-squares difference between simulated and measured signals is minimized.

Using this procedure, we found maximal absorption coefficient changes of $1.85\times 10^{-6}\;{\text{mm}}^{-1}$ at 760 nm and $2.99\times 10^{-4}\;{\text{mm}}^{-1}$ at 850 nm, corresponding to task-related chromophore changes of ΔHbO=1.62 μM and ΔHbR=−0.61μM in the occipital cortex. Since the visual checkerboard stimulus elicits a very strong hemodynamic response, on the order of ∼3% BOLD signal change as measured by fMRI, we scaled down these maximal values by a factor of three to approximate response magnitudes more representative of typical cognitive tasks (Handwerker et al., 2004; Schneider et al., 1993). In the absence of a direct method to estimate task-evoked changes in Δ*μ_a_* across experiments, this scaling provides a pragmatic adjustment to avoid overestimation of simulated signal magnitudes. This resulted in absorption changes of $6.18\;\;\times \;\;10^{-7}\;{\text{mm}}^{-1}$ (760 nm) and $9.95\;\;\times \;\;10^{-5}\;{\text{mm}}^{-1}$ (850 nm), corresponding to ΔHbO=0.54 μM and ΔHbR=−0.20 μM . These values were used as fixed input magnitudes for all subsequent simulations.

### 2.3 Modeling of between-subject variance via simulation of subject-specific cortical sources

Realistic power analyses depend on capturing between-subject variability (Fig. 1B) in both the location and magnitude of the cortical response. We, therefore, simulate a cohort of subject-specific cortical sources with a nominal radius r=7.5 mm (unless otherwise specified) and a canonical center on the grey-matter surface. The radius was chosen as a physiologically plausible approximation of focal activation extent, corresponding closely to approximately half the observed FWHM (≈ 16 mm) of the experimentally reconstructed sources in our dataset.

To model size variability, each subject’s radius *r*^′^ is sampled from a normal distribution N(μ=r,σ=r/6). To model location variability, we shift the cortical source center by an isotropic Gaussian offset N(μ=0,σ=5 mm) and snap it to the nearest grey-matter mesh node. These spatial scales are supported by prior structural and functional neuroimaging studies reporting millimeter-scale between-subject variability in cortical landmarks and source locations (Noyce et al., 2022; Thompson et al., 1996). To model amplitude variability, each subject’s peak Δ*μ_a_* change is drawn from a normal distribution $N(\mu =6.18\times 10^{-7}{\text{mm}}^{-1}\left(\text{760 nm}\right)\text{ or }9.95\times 10^{-5}{\text{mm}}^{-1}$ $\left(\text{850 nm}\right),\;\sigma =\frac{3}{10}\mu )$ and assigned uniformly within the spherical cortical source (zero elsewhere). Finally, each Δ*μ_a_* map is forward-modeled with $J_{\text{spec}}$ to obtain noiseless, channel-wise ΔHbO and ΔHbR signals for each simulated subject. Within-subject noise is added to the channel-level simulated data at a later stage. We repeated this to obtain 1,000 simulated measurements, each representing a unique subject. We computed probabilistic cortical sources by taking the mean across individual cortical sources represented as binary maps on the grey-matter mesh (1 = included, 0 = excluded). Weighted source depth is then calculated as the product of the probabilistic cortical source and each node’s depth from the scalp. We estimated the pathlength of photons that traverse cortical sources using the forward model obtained via NIRFAST. For each channel, tissue-specific pathlengths were computed and weighted by that channel’s pathlength through the probabilistic cortical source. Fractional tissue contributions were obtained by normalizing the weighted tissue-specific pathlengths by the total weighted pathlength.

### 2.4 Estimation of within-subject variance

We quantify within-subject variability as the standard error of channel-wise β coefficient estimates from GLM analyses (Fig. 1C), as a function of block count, enabling assessment of statistical power relative to the number of blocks.

Within-subject variance was estimated via bootstrapping across stimulus block counts b∈{4,8,12,16,20,24}. For each *b*, we generated 100 resamples by randomly selecting *b* blocks of 32.5 s duration (2.5 s baseline, 15 s stimulus, 15 s post-stimulus rest; sampled with replacement) and concatenating them by baseline shifting the beginning of each block to align to the end of the preceding block, thereby preserving temporal continuity. The post-stimulus interval is designed to provide sufficient time for the hemodynamic response to return toward baseline (Colier et al., 2001; Wijeakumar et al., 2012), and any residual low-frequency variation introduced by concatenation is expected to have limited influence on estimation of the task-related regressors. A GLM was then fit to each resampled dataset, and the resulting $\hat{\beta }$ coefficients for ΔHbO were retained.

The standard deviation of $\hat{\beta }$ across resamples, for each subject and channel, provided an empirical standard error ${\text{SE}}_{\hat{\beta }}\left(b\right)$. Averaging ${\text{SE}}_{\hat{\beta }}\left(b\right)$ over subjects and channels, we fitted the inverse-root model:

$${\text{SE}}_{\hat{\beta }}\left(b\right)=\frac{\sigma_{\hat{\beta }}}{\sqrt{b}}$$

where $\sigma_{\hat{\beta }}$ characterizes within-subject estimation variability. This formulation was used for parameterization in subsequent simulations.

### 2.5 Cluster-based permutation test power analyses

We evaluated statistical sensitivity at α=0.05 as a function of sample size (*s*) and block count (*b*) by simulating 1,000 datasets for each combination (Fig. 1D). Each dataset comprises randomly drawn instances—sampled without replacement—of a defined sample size from our pool of 1,000 subjects. To incorporate within-subject variability, we added zero-mean Gaussian noise ($\varepsilon ∼N(\mu =0,\sigma =SE_{\hat{\beta }}(b))$ ) to each channel’s ΔHbO signal, where the noise level was determined by the number of experimental blocks modeled. For each simulated dataset, we applied two-tailed MNE-Python permutation cluster tests (Gramfort et al., 2013; Maris & Oostenveld, 2007) with a sparse adjacency matrix defining neighboring channels. T-statistics were computed for the contrast of interest, and clusters of adjacent significant channels were identified. Across channels within a cluster, t-statistics were summed to give a cluster-level statistic. The condition labels were then shuffled across 1,024 permutations, and the same procedure was followed to identify cluster-level statistics. The most extreme cluster statistic value observed on each permutation was added to a null distribution. Finally, the p-value of each cluster in the original simulation was given by the proportion of this null distribution more extreme than the observed statistic. We defined power as the fraction of the 1,000 simulations in which at least one cluster reached p<0.05 (Fig. 1E). Throughout, we use 80% power as a conventional reference point for sufficient power, rather than a prescriptive threshold (Button et al., 2013). We additionally assessed empirical Type I error under the global null using 10,000 simulation repetitions with 30 blocks and varying subject count, using the same max-cluster permutation procedure as in the primary analyses (Supplementary Fig. S5). A schematic of the cluster-based permutation procedure is provided in Supplementary Figure S2.

### 2.6 Systematic evaluation of cortical source and measurement features

To illustrate our power analysis framework, we ran four simulations exploring the impacts of source depth, source size and spatial separation, cortical region, and channel density. First, we positioned a cortical source in the left auditory cortex at superficial (≈16 mm ), intermediate (≈25 mm ), or deep (≈34 mm ) distance from the scalp and recorded with a temporal-lobe optode montage, illustrating how depth alters signal capture. Next, we measured pairs of sources on superior temporal gyrus (STG) with radii of 5 mm and 7.5 mm at center-to-center distances of 2.5, 5, and 10 mm using the same montage. We assessed cortical location effects by modeling a whole-head dense montage (Fig. 1A) over matched-depth sources in frontal, temporal, occipital, and parietal lobes. Finally, to evaluate how channel density affects power, we applied three temporal-cortex montages over an STG source with cumulative first-, second-, and third-nearest-neighbor (NN) channel configurations.

## 3 Results

### 3.1 Within-subject variance

Within-subject estimation variability followed an inverse-root relationship with block count, yielding an estimated $\sigma_{\hat{\beta }}=0.22\;\mu \text{M}$ ($R^{2}=0.985$ ) for our visual checkerboard dataset. Across datasets from our lab, $\sigma_{\hat{\beta }}$ ranges from 0.20 to 0.25 μM depending on the inclusion of short-channel regressors (derived from short source–detector separations that account for superficial physiology (Gagnon, Cooper, et al., 2012; Zhou et al., 2020)) in our GLM framework. Short channels were not used in our visual checkerboard experiment; for all simulations, we therefore used a value of $\sigma_{\hat{\beta }}=0.20\mu \text{M}$ .

### 3.2 Depth sensitivity

**Fig. 2.**
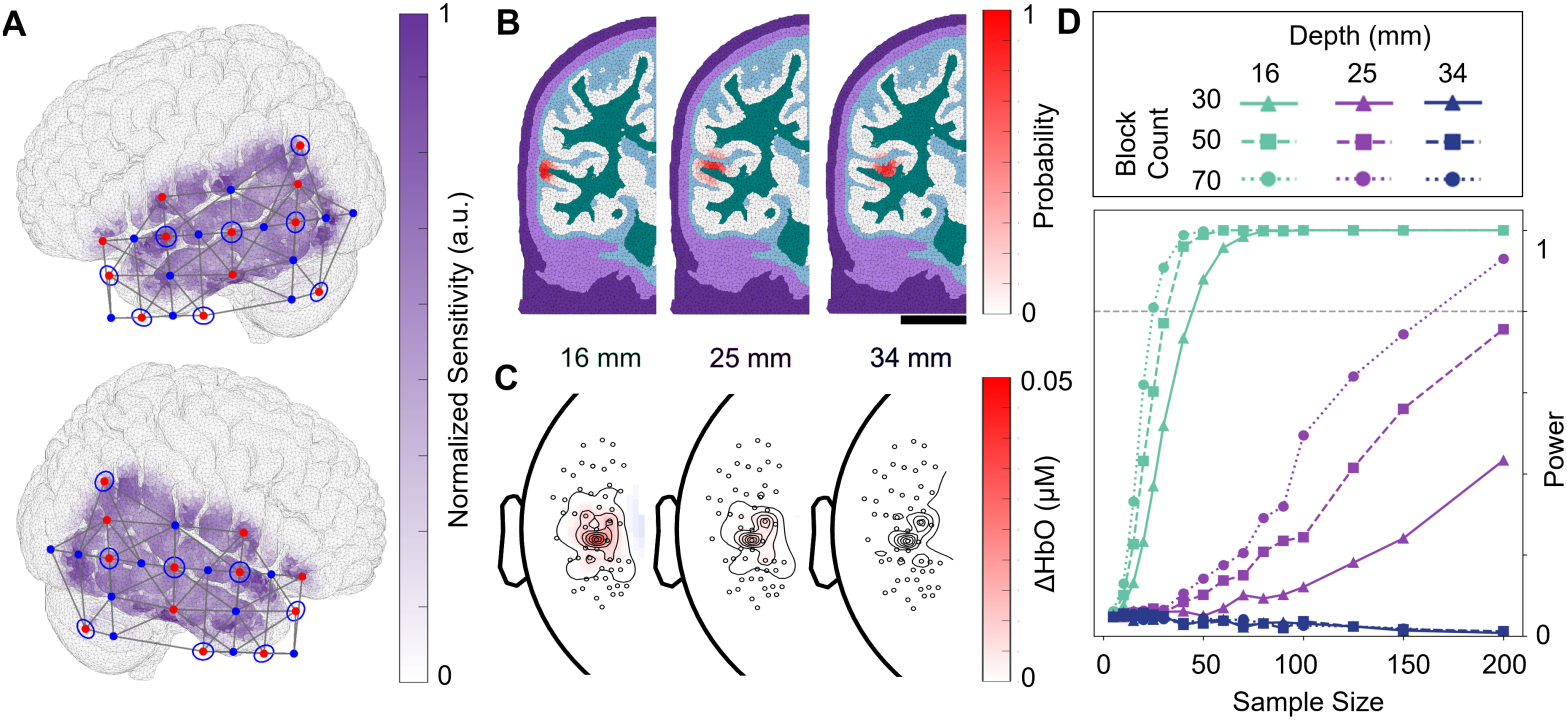
The effect of cortical source depth on predicted channel-level ΔHbO and power analyses. (A) Normalized flatfield sensitivity of the optode montage with bilateral coverage of temporal lobe. Red and blue dots represent source and detector locations respectively. Blue circles surrounding sources represent the locations of short-distance detectors (forming 8 mm channels). Grey lines represent long channels topographically (≥20 mm). (B) Probabilistic locations of simulated cortical sources with different weighted depths from the scalp surface (depths: 16, 25, and 34 mm; scale bar: 40 mm), and (C) their predicted channel-level ΔHbO. Circles denote channel locations. (D) Power estimates as a function of sample size, weighted source depth (16 mm: turquoise, 25 mm: purple, 34 mm: blue) and block count (30 blocks: solid line/triangles, 50 blocks: dashed line/squares, 70 blocks: dotted line/circles) assuming $\sigma_{\hat{\beta }}=0.20\;\mu \text{M}$ . The dashed grey line marks power = 0.8.

Figure 2A shows the optode array and its normalized sensitivity covering the temporal lobe. Source–detector distances range from 14.4–44.9 mm (mean 29.6 mm, SD 9.6 mm). Figure 2B shows probabilistic representations of three cortical sources in the temporal lobe of different depths from the scalp surface (16 mm, 25 mm, 34 mm). The shallowest source is centered on the surface of STG in the temporal cortex, the next source on the bank of lateral sulcus, and the deepest source approaching Heschl’s gyrus. Figure 2C depicts predicted channel-level ΔHbO signals, illustrating the expected exponential decay of channel-level ΔHbO with increasing source depth. Maximal ΔHbO across channels are 0.039 μM (16 mm depth), 0.012 μM (25 mm depth), and $7.28\times 10^{-4}\;\mu \text{M}$ (34 mm depth). Figure 2D illustrates how cluster-based permutation power analyses are affected by source depth, sample size (0 to 200), and block count (30, 50, 70).

Power analyses show that sources at 16 mm depth reach 80% power with as few as 25 subjects and between 30–70 blocks. Sources at 25 mm depth require many more subjects—between 150–200 subjects and 70 blocks is the lowest combination of explored design parameters that achieves the same power threshold. At 34 mm depth, power remains near chance (≈ 5%) regardless of sample size or block count.

### 3.3 Spatial separation and size

**Fig. 3.**
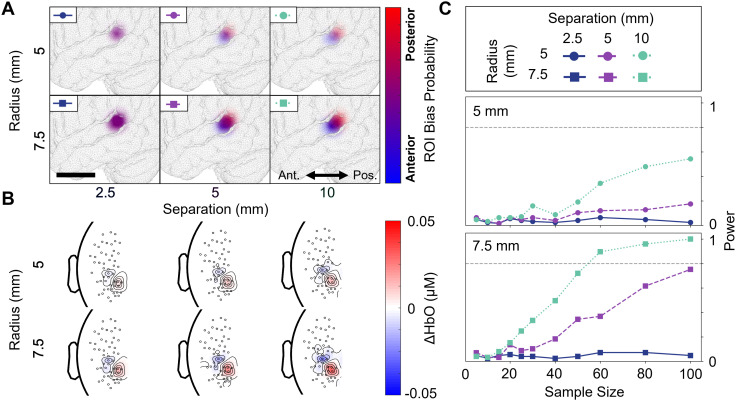
The effect of radius and separation between contrasted left hemisphere cortical sources on predicted channel-level ΔHbO and power analyses. (A) Probabilistic maps of contrasted cortical sources varying in size (radius: 5 mm, 7.5 mm) and spatial separation (2.5 mm, 5 mm, 10 mm) located along the surface of left posterior STG (scale bar: 40 mm). Colors correspond to the probability a given brain mesh node is included in the posterior cortical source (red), the anterior cortical source (blue), both (purple; hue is determined by relative contribution of each source); or neither (white). Insets correspond to the data in (C) given a specific radius / spatial separation combination. (B) Contrast in predicted posterior versus anterior channel-level ΔHbO. (C) Power estimates as a function of sample size and separation between cortical sources, for radius = 5 mm (top) and radius = 7.5 mm (bottom), assuming $\sigma_{\hat{\beta }}=0.20\;\mu \text{M}$ , and n = 40 blocks. The dashed grey lines mark power = 0.8.

Figure 3A shows probabilistic representations of contrasted cortical sources both of the same size (radius: 5 mm, 7.5 mm), separated on the surface of left posterior STG (2.5 mm, 5 mm, 10 mm). Differences in the distance from the scalp for source pairs in all contrasts were small (range: 0.07–0.50 mm). Predicted channel-level ΔHbO for each is shown in Figure 3B, modeled using the same optode montage as Section 3.2. Corresponding power analysis results are shown in Figure 3C, assuming 40 measurement blocks per subject. Contrasts between 5 mm-radius cortical sources separated by 2.5, 5 and 10 mm produced respective maximum ΔHbO across channels of 0.005μM , 0.008μM , and 0.010μM , and would require well over 100 subjects, even at 10 mm separation, to achieve 80% power. For the larger, 7.5 mm-radius sources, respective maximum ΔHbO across channels increased to 0.012μM , 0.018μM and 0.025μM ; 80% power was achieved with 50–60 subjects at 10 mm separation, nearly reached with 100 subjects at 5 mm separation, and remained at chance levels for 2.5 mm separation.

### 3.4 Cortical location

**Fig. 4.**
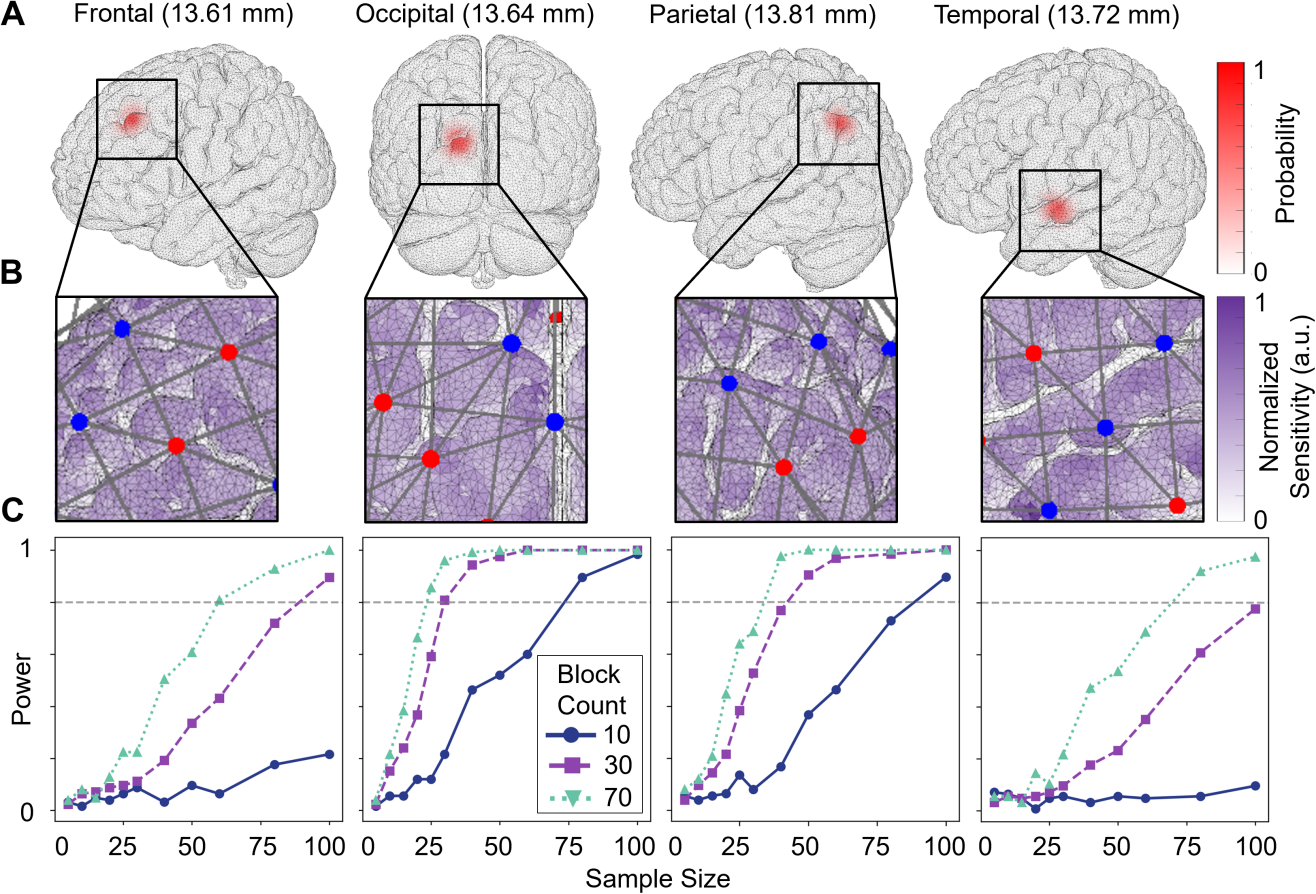
The effect of brain region on statistical power. (A) Probabilistic representations of four cortical sources located in frontal, occipital, parietal and temporal lobes. Weighted depths from the scalp are included in parentheses. Boxes represent regions shown in Panel B. (B) Normalized flatfield sensitivity (purple) and optical sources (red), detectors (blue), and channels (grey lines) over boxed regions surrounding each source. (C) Power estimates for each source as a function of sample size (0–100) and block count (10, 30, 70), $\sigma_{\hat{\beta }}=0.20\mu \text{M}$ . The dashed grey lines mark power = 0.8.

We investigate how cortical sources at similar depths from the scalp affect power curves, even when size and magnitude of the focal activation are held constant. Figure 4A illustrates simulated cortical sources in frontal, occipital, parietal, and temporal lobes, with respective depths from the scalp of 13.61, 13.64, 13.81, and 13.72 mm. Figure 4B depicts the modeled optode montage, channels, and flatfield sensitivity over the cortical regions shown in Figure 4A. Source–detector distances range from 15.0–44.9 mm (mean 32.6 mm, SD 6.7 mm). Although all simulations use the same dense whole-head montage, channel coverage differs across regions: frontal sources are sampled by two channels with overlapping high-sensitivity regions at the source location, occipital sources by four channels, parietal sources by three channels, and temporal sources by six channels; however, the temporal source is positioned directly beneath a detector and does not fall within the region of maximal sensitivity for any individual channel. Maximal group-averaged channel-level ΔHbO predicted from each source is highest for the parietal and occipital sources (0.037μM and 0.036μM ), followed by the frontal and temporal sources (0.025μM and 0.022μM ). Channel layouts and distributions of predicted ΔHbO for each cortical source are shown in Supplementary Figure S3.

Figure 4C plots power estimates for each modeled source as a function of sample size (0–100 subjects) and block count (10, 30, 70). The occipital source yields the strongest power curves, despite a marginally lower peak ΔHbO than the parietal source. The 80% power threshold is exceeded at approximately 20–25, 25–30, and 60–80 subjects for block counts of 70, 30, and 10, respectively. The parietal source shows the next strongest power, crossing the same threshold at roughly 30–35, 35–50, and 80–100 subjects. In contrast, the frontal source exceeds 80% power only at approximately 60 subjects for 70 blocks and 80–100 subjects for 30 blocks, while the temporal source crosses the threshold at 60–80 subjects for 70 blocks and remains below it at 100 subjects for 30 blocks. For both frontal and temporal sources, power for 10 blocks does not meaningfully increase above chance.

These channel-level magnitudes and power analysis results can be compared to fractional pathlengths observed by photons that reach each cortical source. Table 2 shows the fractional contributions of each tissue layer based on a weighted average of each channel’s tissue fractions. For the cortical sources we selected, fractional grey matter contributions across the sources ascend from 7.2% in the parietal region to 8.3% in the frontal, 9.8% in the occipital, and 10.3% in the temporal region.

**Table 2.** Estimated fractional pathlength of photons reaching cortical sources.

Source	Scalp	Skull	CSF	Grey matter	White matter
Frontal	29.7%	43.6%	18.1%	8.3%	0.4%
Occipital	24.3%	44.3%	20.4%	9.8%	1.3%
Parietal	37.9%	39.1%	15.3%	7.2%	0.4%
Temporal	28.9%	42.0%	18.1%	10.3%	0.7%

### 3.5 Channel density

**Fig. 5.**
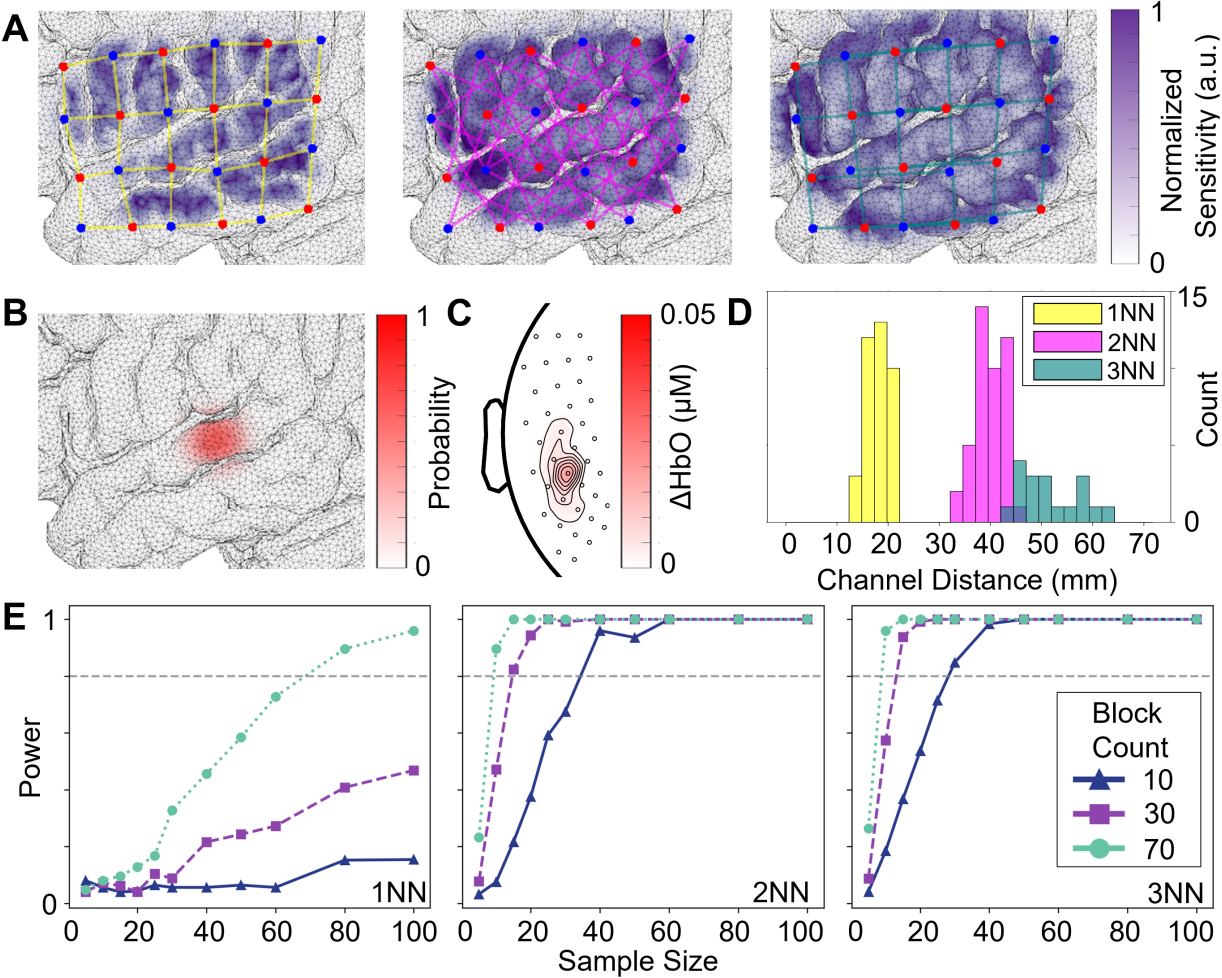
The effect of optode montage channel density on statistical power. (A) Flatfield sensitivity maps of the 1NN to 3NN optode montages covering temporal lobe. Yellow lines represent 1NN channels, magenta lines represent 2NN channels, and teal lines represent 3NN channels. For visualization, channels in lower density montages are excluded from the depictions of higher density montages. 3NN channels appear to overlap with 1NN channels as they lie along the same axes, but are nearly 3 × longer. (B) Probabilistic map of modeled cortical source on STG. (C) Predicted channel-level ΔHbO for the 1NN montage. (D) Histogram of channel distances per montage. (E) Power estimates as a function of sample size and block count, assuming $\sigma_{\hat{\beta }}=0.20\;\mu \text{M}$ . The dashed grey lines mark power = 0.8.

Figure 5 illustrates how channel density for a static source-detector layout over the temporal cortex impacts sensitivity and statistical power. Flatfield sensitivity profiles deepen with increased channel density and length, as seen in Figure 5A. Channels from lower NN optode montages are cumulatively included in higher ones, so the first-nearest neighbor (1NN) montage includes only the yellow channels of Figure 5A, the second-nearest neighbor (2NN) includes both the yellow and magenta channels, and the third-nearest neighbor (3NN) all three sets of channels. Figure 5B shows the probabilistic map (across-subject mean) of the STG source, located beneath each optode array. An example channel-level ΔHbO map (Fig. 5C) for the 1NN array peaks at roughly 0.018 µM. Maximum channel-level ΔHbO increases two-fold and plateaus for greater kNN montages. Predicted channel-level ΔHbO maps for all three nearest-neighbor montages (1NN–3NN) are provided in Supplementary Figure S4.

Figure 5D shows histograms of channel distances for the original 1NN montage (yellow), the channels added in the 2NN montage (magenta), and those added in the 3NN montage (teal). Mean distance increases progressively from 17.9 ± 2.3 mm in the 1NN montage to 40.0 ± 3.0 mm for the 2NN additions and 51.6 ± 5.7 mm for the 3NN additions. Power curves shown in Figure 5E reveal that 1NN is underpowered with high but realistically obtainable experimental design parameters (requiring ≈ 70 blocks and ≈ 70 subjects to reach 80% power), whereas 2NN achieves 80% power with fewer than 40 subjects at lower block counts (10–30). Moving to 3NN and higher adds minimal further benefit in power.

## 4 Discussion

We present a methodological framework for predicting channel-level fNIRS/DOT measurements from a target cortical source and an estimated Δ*μ_a_*, addressing the challenge of high measurement variability and limited guidance for *a priori* power analyses. Our pipeline integrates a realistic anatomical model, empirically informed absorption magnitude estimation, within- and between-subject variance estimation, task-specific effects on optical properties, and cluster-based permutation testing. Across four simulations, we demonstrate how anatomical and measurement features influence channel-level signal magnitude and statistical power. Our approach enables researchers to explore how sample size and experiment design affect the feasibility of measuring signals from specific cortical targets, without relying on retrospective or ad hoc analyses.

As with other noninvasive neuroimaging techniques, fNIRS/DOT measurements are often noisy, primarily due to physiological confounds, anatomical variation, and aspects of experimental setup (e.g., optode placement, contact quality, susceptibility to motion artifacts). As a result, many researchers remain uncertain about whether these methods are appropriate for their specific research questions. While past work has made important progress in characterizing sensitivity (Ferradal et al., 2014; Gagnon, Yücel, et al., 2012; Strangman et al., 2002; Zhai et al., 2020; Zimeo Morais et al., 2018; Zinos et al., 2024), improving spatial resolution (Cui et al., 2011; Doulgerakis et al., 2019; Eggebrecht et al., 2012; Markow et al., 2025; White & Culver, 2010), and applying methodological advancements (Cao et al., 2023; Eggebrecht et al., 2014; Hassanpour et al., 2015; Schroeder et al., 2023), these efforts have typically stopped short of translating physical limitations into concrete design guidance. Our approach builds upon this foundation by expressing feasibility in terms of sample size and block count, offering a pathway for making principled design decisions at the planning stage. Importantly, the power estimates presented here are intended for group-level inference and do not preclude robust task-evoked responses at the single-subject level, as has been demonstrated in prior fNIRS/DOT studies (Sinfield et al., 2025; Zeff et al., 2007).

To enable predictions of channel–level measurements, we first estimated absorption coefficient changes during visual checkerboard stimulation that correspond to a maximal ΔHbO of 1.62 μM (≈ 1.10 μM within the FWHM ellipsoid). These values are slightly higher than those previously reported in frequency–domain NIRS (FD–NIRS) studies (McIntosh et al., 2010; Wijeakumar et al., 2012), possibly due to our higher channel density, sample–dependent variation in optical pathlength, or other experimental factors (Wijeakumar et al., 2012). Absorption magnitudes estimated empirically are not ground truth; inaccuracies could arise due to dataset–specific variability. Further sources of variation in our modeled sources should be kept in mind during interpretation. For instance, regularization–sensitive reconstruction approaches can be accurate, but may still inflate apparent source size and thus underestimate true peaks (Dehghani et al., 2009). Our uniform application of the point–max across a fixed–radius sphere both overstates focal absorption and omits the Gaussian tail, which captures a substantial fraction of the integrated signal. Our absorption estimates and source models could therefore be refined further using high–density DOT (Eggebrecht et al., 2012) and more physiologically realistic source distributions (Dunn et al., 2005; Vazquez et al., 2014).

Our estimations of within-subject variability closely follow an inverse-root relationship, allowing us to parameterize power simulations according to block count. Within-subject variability can be estimated from existing data or a small pilot experiment. When paired with an expected task-evoked amplitude, this yields an approximate effect size that can be used in conventional power calculations (e.g., one-sample *t*-tests) to guide initial sample size and block count decisions. However, the estimated $\sigma_{\hat{\beta }}$ depends on task properties, preprocessing choices, and experimental assumptions, and should not be treated as a universal quantity. Moreover, such simplified calculations do not account for probe coverage or contiguity, cluster-based inference, or multiple-comparisons burden; accordingly, the full simulation framework remains recommended for design-level decisions.

Our simulations show that feasibility in fNIRS/DOT is strongly dependent on anatomical and measurement factors. Our first simulation demonstrated that detecting superficial sources (≈ 16 mm) required fewer than 30 subjects under typical conditions, while deeper regions (≥ 25 mm) were practically undetectable. These results reveal clear trade-offs in experimental design; each design parameter experiences diminishing returns in power. Intermediate-depth sources (e.g., on sulcal banks) illustrate how the choice of sample size and block count can determine whether a cortical source is detectable. Source size also influences detectability, since fNIRS/DOT integrates optical changes over volume and larger targets typically produce stronger signals. Our simulations demonstrated that smaller cortical foci (≤5 mm radius) or closely spaced sources (≤5 mm separation) dramatically increased the sample size and block count required for detection or discrimination—a 50% increase in radius yields greater than 2× the signal strength and allows for sufficient power at realistic sample sizes and block counts (55 subjects; 40 blocks). Together, these findings provide an alternative perspective on spatial resolution by characterizing it in terms of experimental requirements. In such scenarios where design choices may determine feasibility, *a priori* power analyses prove useful.

We explored sources of similar superficial depth across cortical locations and observed marked differences in detectability and statistical power. Frontal and temporal sources were hardest to detect, followed by parietal sources, while occipital sources exhibited the strongest power despite a marginally lower peak ΔHbO than the parietal source. Notably, the parietal source showed higher power than frontal and temporal sources despite relatively low fractional pathlength through grey matter. This contrast underscores that forward-sensitivity metrics are not the sole predictors of statistical power. The observed variation in detectability and statistical power may reflect a combination of factors, including within-subject variability in tissue volumes that alters absorption and scattering (Perdue et al., 2012), between-subject variability in source depth that can be modulated by cortical geometry, and differences in channel coverage and spatial contiguity across regions. Figure 4B illustrates these coverage differences, with occipital sources sampled by a greater number of channels centered near the source, which may favor larger cluster statistics (Strangman et al., 2003), whereas other regions are sampled by fewer or more peripherally positioned channels. The lobe-specific differences shown here emphasize that characteristics of a given target location and the associated optode coverage interact to shape statistical power.

Optical channel density was equally consequential: increasing channel density from 1NN to 2NN nearly doubled sensitivity and reduced the number of required subjects for 80% power from ≈ 70 to under 40. This result is consistent with prior work showing that increased channel density can yield marked improvements in cortical sensitivity and statistical significance (Anderson et al., 2025; Dehghani et al., 2009; Doulgerakis et al., 2019; Habermehl et al., 2012). Our findings complement our earlier analyses that varied source size, separation, and cortical location, underscoring that numerous factors interact to shape signal recovery and statistical power. Together, these results show that assumptions of uniform sensitivity or fixed sample sizes are insufficient for experimental planning. Instead, power depends critically on what is measured, where it is measured, and how it is measured, reinforcing that physical constraints and measurement choices must be evaluated in the context of specific research questions to inform realistic and effective study design.

We did not explicitly test effects of the task-evoked absorption magnitude, instead holding it constant across all simulations. Absorption coefficient changes are not readily available for most functional paradigms. Ideally, subject-specific head models and prior data would be used to compute more accurate Δ*μ_a_* estimates. Δ*μ_a_* can be estimated without the need for task-specific experimental data by assuming a linear relationship between Δ*μ_a_* and BOLD signal changes, allowing us to draw on the larger body of fMRI literature. Our absorption magnitude estimation yielded maximal absorption changes of $1.85\times 10^{-6}\;{\text{mm}}^{-1}$ at 760 nm and $2.99\times 10^{-4}\;{\text{mm}}^{-1}$ at 850 nm for visual checkerboard stimulation. fMRI results suggest such stimuli typically yield on the order of 3% BOLD signal change (Handwerker et al., 2004; Schneider et al., 1993). To extrapolate to a different task of interest with an *x*% BOLD signal change reported, one can scale our Δ*μ_a_* by *x*/3, thus enabling *a priori* power analyses for any task where previously reported fMRI BOLD signals can serve as a useful point of comparison. While the assumption of linear scaling is unlikely to be strictly true (Pierro et al., 2014), this provides a reasonable approximation in the absence of existing fNIRS/DOT measurements.

Our results should be viewed as practical guidelines rather than exact predictions, given the simplifying assumptions in our approach. We relied on a single template head model, which can only approximate individual anatomical differences in tissue volume, head geometry, and differential pathlength factors. Because pathlengths were derived from a standard head model, reporting hemoglobin changes instead of raw optical density changes introduces variance by not accounting for subject-specific pathlength differences. A number of modeling and analysis choices could also be refined. For instance, we assume that measurement noise, motion artifacts, and physiological confounds (cardiac pulsation, respiration, Mayer waves, etc.) are captured by our within-subject β-variance rather than modeled explicitly. Similarly, between-subject variance is computed from a single head model and parameterized based on past literature, which may not generalize to all experimental designs. We also assumed a fixed ≈ 15 s block length; shorter or more complex designs would alter signal magnitude.

Although our framework employs a robust cluster-based permutation test, increasing the number of simulation iterations could further improve the precision of the predicted power estimates, while nominal Type I error control is determined by the cluster-based permutation procedure. Alternative statistical approaches, such as linear mixed-effects models or less conservative methods like Sequential Bayes Factors, may offer complementary insights into study feasibility (Luke et al., 2021; Schönbrodt et al., 2017). Related work performs source-level statistics using fMRI-style random-field cluster analysis (Hassanpour et al., 2014). Our approach could adopt similar methods for faster computation via analytic thresholds, with similar or slightly higher power when data are sufficiently smoothed and sample sizes are adequate. Future work could refine both the anatomical and analysis models by incorporating subject-specific head geometries and DPFs, explicit noise sources, variable block designs, and complementary statistical frameworks.

Beyond modeling choices, the accuracy of our approach can be affected by cohort factors. Aging and many pathologies modify tissue layers (e.g., CSF expansion, cortical thinning) and optical properties, while medications and disease can alter neurovascular coupling and systemic physiology, changing hemodynamic response amplitude/latency and noise structure. Such changes may weaken assumptions of linearity or stationarity in our GLM, or permutation exchangeability in our statistical testing. Accordingly, our recommendations are most reliable for healthy adult populations under dense, contiguous coverage; clinical applications should incorporate study-specific calibration (optical properties/DPFs), small multi-subject pilots to characterize between-subject dispersion, and, when possible, subject-specific head models.

To implement our framework, we recommend following these steps: (1) establish an anatomical head model; (2) design a high-density optode montage optimized for target regions; (3) compute forward sensitivity matrices using tools like NIRFAST (Dehghani et al., 2008); (4) draw from experimental data or literature to define a target grey matter source with predicted Δ*μ_a_* values; (5) estimate within-subject variance in the task predictor using either experimental data or the value reported here for GLM analysis (i.e., 0.2 *μ*M); (6) perform cluster-based permutation testing on simulated datasets to assess power of a given design. We provide online coding resources to support adoption of this approach.

Taken together, our results show that anatomical and measurement-related factors—such as depth, spatial resolution, source size, source location, and channel density—have dramatic effects on statistical power in fNIRS and DOT. By linking these factors to sample size and block count, our framework enables task- and source-specific feasibility assessments beyond rule-of-thumb heuristics. Building on prior modeling efforts, this quantitative, *a priori* approach to feasibility assessment empowers researchers to design well-powered studies, improving the rigor and efficiency of fNIRS/DOT research.

## Supplementary Material

Supplementary Material

## Data Availability

User-friendly code to enable adoption of our approach is provided as an open-source MATLAB and Python software package at https://github.com/esbulger/fnirs-power.
