# Supplementary material for: Measurement prediction and power analysis for fNIRS and DOT
Source: Imaging Neurosci (Camb). 2026 Jul 1;4:IMAG.a.1289. doi: 10.1162/IMAG.a.1289 (PMC13326666; doi:10.1162/IMAG.a.1289)
Supplement: Supplementary Material [file IMAG.a.1289_supp.pdf]

Supplementary Materials for *Measurement Prediction and Power Analysis for fNIRS and DOT*

Eli Bulger[1], Jiaming Cao[1], Abigail L. Noyce[3,4], Barbara G. Shinn-Cunningham[1,2,3,4], Jana M. Kainerstorfer[1,2,3*]

[1] *Department of Biomedical Engineering, Carnegie Mellon University, Pittsburgh, Pennsylvania, United States*

[2] *Department of Electrical and Computer Engineering, Carnegie Mellon University, Pittsburgh, Pennsylvania, United States*

[3] *Neuroscience Institute, Carnegie Mellon University, Pittsburgh, Pennsylvania, United States*

[4] *Department of Psychology, Carnegie Mellon University, Pittsburgh, Pennsylvania, United States*

[*] Correspondence: jkainers@andrew.cmu.edu

Contents

I Supplementary Methods **3**

Figure S1: Experimental dataset and processing steps used to estimate absorption magnitude 4

Figure S2: Cluster-based permutation test power analysis workflow 6

II Supplementary Results **7**

Figure S3: Predicted ΔHbO distributions for cortical sources in Section 3.4 7

Figure S4: Predicted ΔHbO distributions for 1-3NN channel layouts in Section 3.5 8

Figure S5: Empirical Type I error from 10,000 null simulations 8

I Supplementary Methods

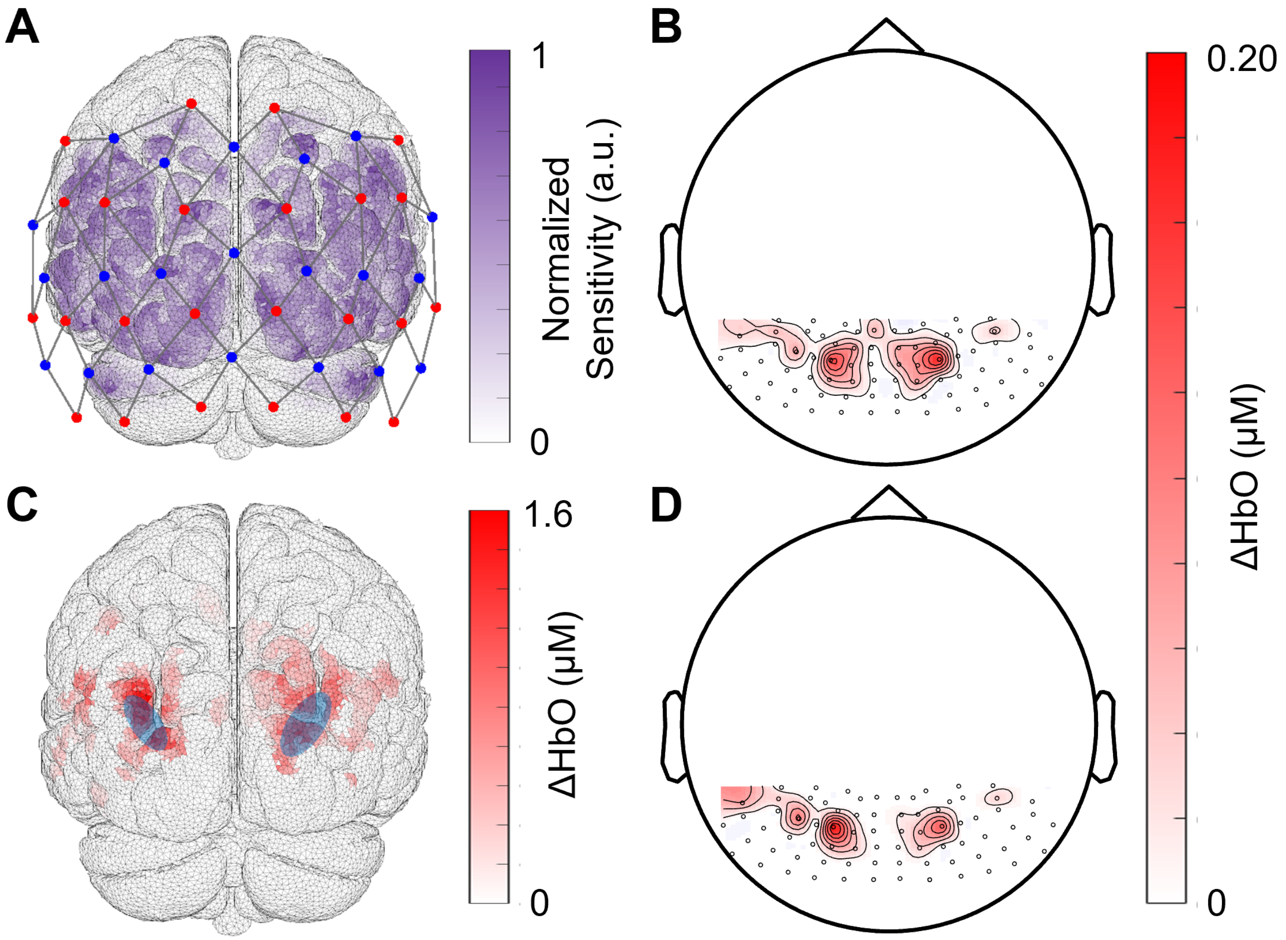

Figure S1. Experimental dataset and processing steps used to estimate absorption magnitude. (A) Normalized flat–field sensitivity profiles (purple) for the dense occipital montage, shown with optical sources (red), detectors (blue), and source–detector channels (gray lines). (B) Measured group–averaged channel–level ΔHbO responses computed from GLM β coefficients; channels are masked at $p < 0.05$. (C) Group–averaged cortical ΔHbO source reconstruction on measured data, computed from the reconstructed $\Delta\mu_a(760\,\mathrm{nm})$ and $\Delta\mu_a(850\,\mathrm{nm})$ maps using spectral unmixing with molar extinction coefficients; cortical nodes are masked at $p < 0.05$. The full-width half-maximum (FWHM) extents of Gaussian ellipsoids fitted to the data are overlaid in blue. (D) Predicted channel–level ΔHbO generated by forward modeling the reconstructed sources in (C); channels are masked at $p < 0.05$. The absorption magnitude was estimated by scaling the reconstructed $\Delta\mu_a$ maps used to compute (C) such that the predicted channel–level responses in (D) best matched the measured responses in (B) using least–squares minimization.

Empirical half-maximum FWHM estimation

To summarize focal activation size, we estimate a Gaussian ellipsoid for each hemisphere from the reconstructed 850 nm $\Delta\mu_a$ map. This approach provides a stable description of focal extent and a corresponding region of interest for averaging ΔHbO and ΔHbR. In this context, FWHM serves as a descriptive reference for focal size rather than as a more commonly reported measure of DOT spatial resolution (White & Culver, 2010).

Let $\mathbf{x}_i \in \mathbb{R}^3$ denote the coordinates of grey-matter nodes, with corresponding reconstructed $\Delta\mu_a$ amplitudes a_i. For each hemisphere, the peak magnitude is

$$a_{\max} = \max_i |a_i|, \tag{S1}$$

and the empirical half-maximum region is the set of nodes satisfying

$$|a_i| \geq 0.5\, a_{\max}. \tag{S2}$$

Using only these half-maximum nodes, we compute a weighted centroid and weighted covariance. The weights are

$$w_i = \left(\frac{|a_i|}{a_{\max}}\right)^2. \tag{S3}$$

This weighting gives greater influence to nodes with stronger reconstructed signal. The weighted centroid is

$$\boldsymbol{\mu} = \frac{\sum_i w_i \mathbf{x}_i}{\sum_i w_i}, \tag{S4}$$

and the weighted covariance matrix is

$$\boldsymbol{\Sigma}_{\text{raw}} = \frac{\sum_i w_i (\mathbf{x}_i - \boldsymbol{\mu})(\mathbf{x}_i - \boldsymbol{\mu})^\top}{\sum_i w_i}. \tag{S5}$$

The eigenvectors of $\boldsymbol{\Sigma}_{\text{raw}}$ define the principal axes of the focal response. Let $\mathbf{V}$ denote these eigenvectors, ordered by decreasing eigenvalue. The half-maximum nodes are then projected onto these axes according to

$$\mathbf{y}_i = \mathbf{V}^\top (\mathbf{x}_i - \boldsymbol{\mu}). \tag{S6}$$

For each principal axis $j \in \{1, 2, 3\}$, the half-width is defined from the 5th and 95th percentiles of the

projected coordinates,

$$r_j = \frac{Q_{95}(y_j) - Q_5(y_j)}{2}, \tag{S7}$$

and the corresponding full width is

$$\mathrm{FWHM}_j = 2r_j. \tag{S8}$$

Using percentiles rather than the extreme projected values is necessary to reduce the influence of isolated outlying nodes in this experimental dataset.

Focal size is summarized by the root-mean-square of the three principal-axis widths,

$$\mathrm{FWHM} = \sqrt{\frac{\mathrm{FWHM}_1^2 + \mathrm{FWHM}_2^2 + \mathrm{FWHM}_3^2}{3}}. \tag{S9}$$

These widths define a Gaussian FWHM ellipsoid centered at $\boldsymbol{\mu}$.

Finally, mean ΔHbO and ΔHbR are computed within this FWHM ellipsoid. This ellipsoid provides a consistent focal region for summarizing the hemodynamic response and for comparison with previously reported visual-cortex DOT values.

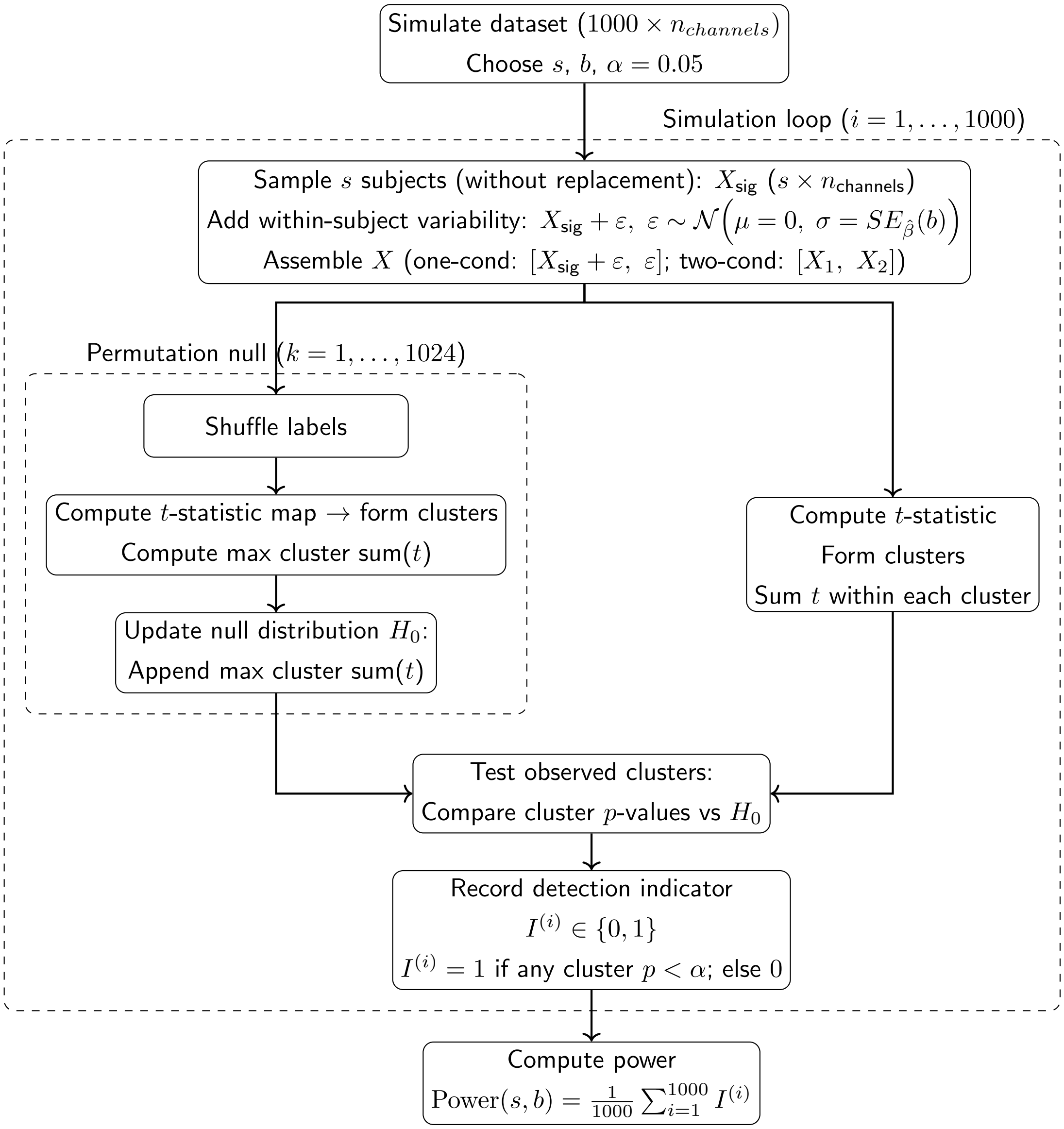

Figure S2. Schematic of the cluster-based permutation procedure used for power estimation. For each sample size s and block count b, a simulation loop $(i = 1, \ldots, 1000)$, upon each iteration, samples s subjects without replacement and adds within-subject noise scaled by $\text{SE}_{\hat{\beta}}(b)$ to form the test input X. Each iteration then follows two parallel branches. In the left (null) branch, a permutation null distribution is built by shuffling condition labels ($k = 1, \ldots, 1024$), recomputing channel-level statistics, forming spatial clusters using the adjacency matrix, and storing the maximum cluster statistic from each permutation to form H_0. In the right (observed) branch, the same statistical and clustering steps are applied to the unpermuted data. Observed clusters are compared against H_0 to compute cluster-level p-values, and power at (s, b) is defined as the fraction of simulations in which at least one cluster reaches $p < \alpha$.

II Supplementary Results

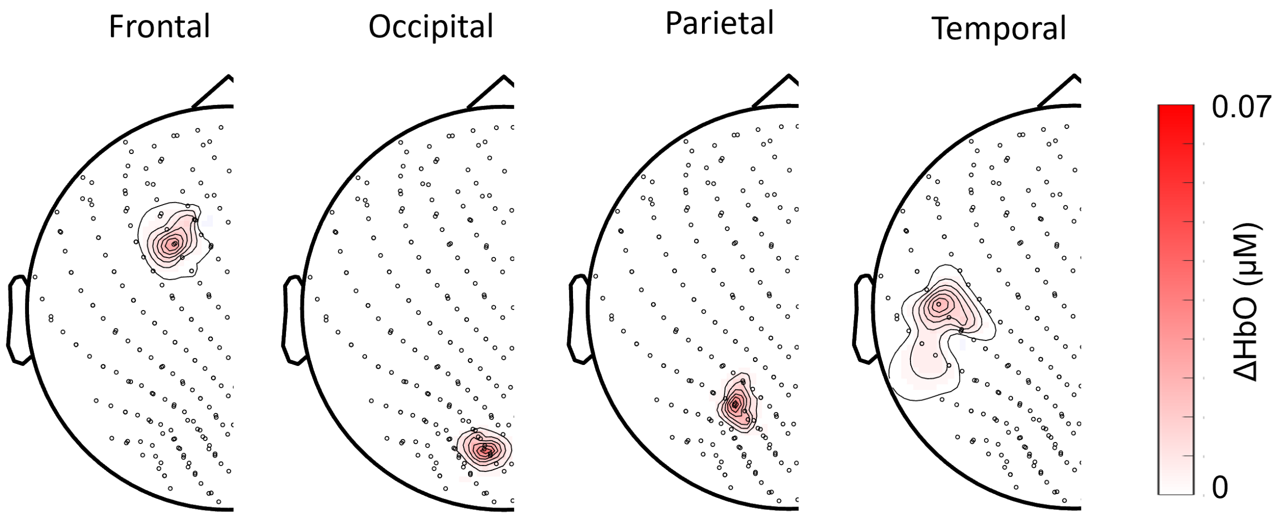

Figure S3. Predicted channel-level ΔHbO for the (from left to right) frontal, occipital, parietal and temporal cortical sources used in Section 3.4. Each circle illustrates the midpoint of a channel in the optode montage.

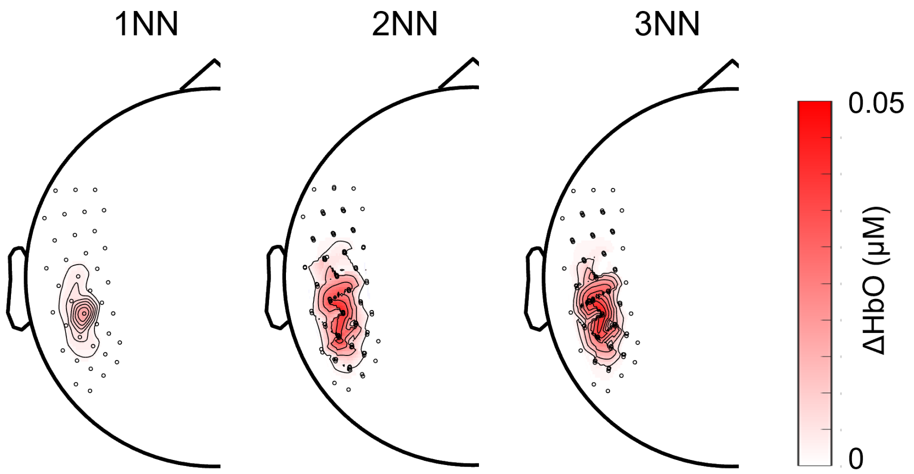

Figure S4. Predicted channel-level ΔHbO for cumulative 1NN (left), 2NN (middle) and 3NN (right) channel layouts in Section 3.5. Each circle illustrates the midpoint of a channel in the optode montage.

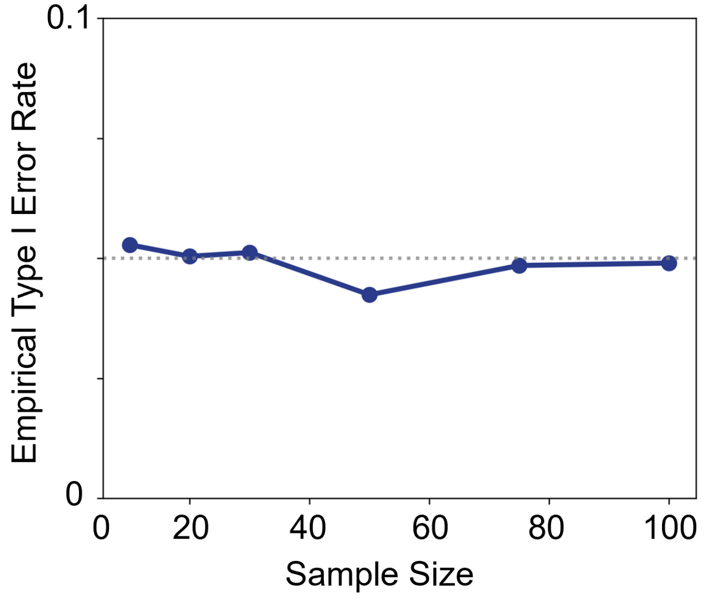

Figure S5. Empirical family-wise Type I error rate estimated from 10,000 null simulation repetitions using the cluster-based permutation procedure, shown as a function of subject count with block count fixed at 30. The dotted grey line marks the nominal $\alpha = 0.05$ level.

References

White, B. R., & Culver, J. P. (2010). Quantitative evaluation of high-density diffuse optical tomography: In vivo resolution and mapping performance. *https://doi.org/10.1117/1.3368999*, *15*(2), 026006. https://doi.org/10.1117/1.3368999
